# Does Breastfeeding Protect Against Psychosis? Findings from a Case–Control Study

**DOI:** 10.1192/j.eurpsy.2026.11841

**Published:** 2026-07-31

**Authors:** R. Palacios-Garrán, D. Gonzalez Mora, N. Laherrán Cantera, M. J. Sánchez González

**Affiliations:** 1 Mental Health Unit, Hospital Universitario Jerez de la Frontera; 2 Obstetrics and Gynaecology Unit, Hospital Universitario Jerez de la Frontera, Jerez de la Frontera, Spain

## Abstract

**Introduction:**

Breastfeeding provides multiple short- and long-term health benefits, including reduced infections, improved neurodevelopment, and possibly lower risk of neuropsychiatric disorders. However, its relationship with psychosis remains unclear. Prior evidence is limited, inconsistent, and focused mainly on schizophrenia, with most studies failing to detect an association. Evidence on other psychotic disorders is virtually absent.

**Objectives:**

This study aimed to examine the association between breastfeeding and the risk of first-episode psychosis (FEP) compared with healthy community control and also explore differences in perinatal variables (mode of delivery, birth weight, and Apgar scores).

**Methods:**

We conducted a cross-sectional case–control study including 26 outpatients with incident FEP (aged 18–40 years, recruited from the Hospital de Jerez early intervention program between January 2023 and December 2024) and 26 healthy controls from the same catchment area. Exclusion criteria were intellectual disability, medical conditions, or substance dependence. Breastfeeding history and perinatal variables were collected via caregiver questionnaires. Statistical analyses included Chi-square/Fisher’s exact tests and Mantel’s linear-by-linear association for trend.

**Results:**

Participants with FEP (mean age 24.7, SD 6.8; 23.1% female) and controls (mean age 26.1, SD 4.1; 42.3% female) did not differ in delivery mode, birth weight, or Apgar scores. However, marked differences were observed in breastfeeding: 53.8% of FEP vs 92.3% of controls had been breastfed (χ²=9.586, p=0.002), corresponding to lower odds of FEP among breastfed individuals (OR=0.10, 95% CI=0.01–0.56). A medium–large effect size was observed (φ=0.434). Duration of breastfeeding also differed across ordered categories (χ²=7.576, p=0.006), with controls showing progressively longer durations. Linear trend analysis confirmed a graded pattern (χ²=5.55, p=0.019).

**Image 1:**
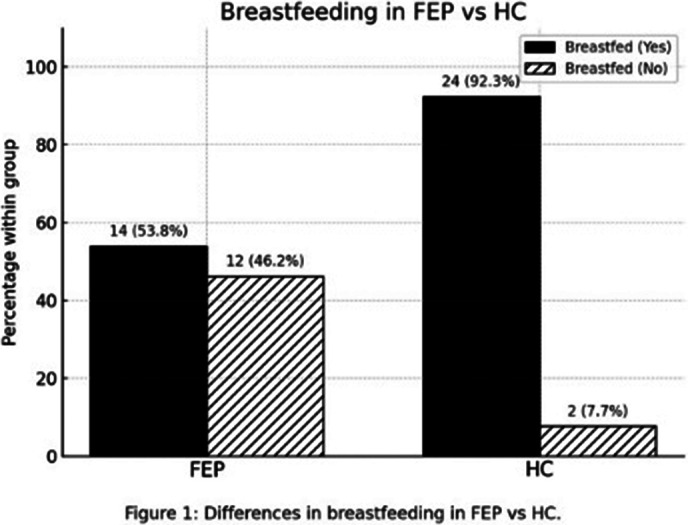

**Conclusions:**

Our findings suggest that breastfeeding may exert a protective effect against the development of FEP, with both presence and duration of breastfeeding inversely associated with psychosis risk. While causality cannot be inferred and potential confounders remain, these results align with previous evidence linking shorter breastfeeding with increased schizophrenia risk. Biological mechanisms (immune modulation, neurodevelopmental support, microbiota shaping) and psychosocial factors (mother–infant bonding) may underlie this association. Larger prospective studies are warranted to confirm these findings and to clarify breastfeeding’s potential role in psychosis prevention.

**Disclosure of Interest:**

None Declared

